# Endonasal dacryocystorhinostomy in children

**DOI:** 10.1016/S1808-8694(15)31239-8

**Published:** 2015-10-20

**Authors:** Denis Knijnik

**Affiliations:** Otorhinolaryngologist, Division of palpebral and lacrimal surgery. Hospital Petrópolis, Porto Alegre

**Keywords:** dacryocystorhinostomy, lacrimal apparatus diseases, lacrimal duct obstruction, endoscopy, child, child, preschool

## Abstract

**Aim:**

To verify whether our results with endonasal endoscopic dacryocystorhinostomy in children with nasolacrimal duct obstruction allow us to consider this technique a valid treatment alternative for children.

**Study design:**

clinical with transversal cohort.

**Material and Method:**

Twenty-seven endoscopic endonasal dacryocystorhinostomies were performed in children 2 to 12 years of age for nasolacrimal duct obstruction. Previous probings in all patients were unsuccessful. The technique employed uncinectomy and a small lacrimal sac opening. Follow-up time was 3 months.

**Results:**

Twenty-one surgeries (77,8%) were successful. The only complication was silicone prolapse in one case.

**Conclusion:**

Our results confirm endoscopic endonasal dacryocystorhinostomy as an acceptable and safe method for treating children with nasolacrimal duct obstructions that are resistant to probings.

## INTRODUCTION

Congenital obstruction of the nasolacrimal duct produces persistent secretion and epiphora (excessive tearing) in children. Treatment with single stent leads to excellent outcomes in simple obstructions of Hasner valve; on the contrary, it is highly unsuccessful in obstructions at the lacrimal sac-lacrimal duct intersection, which is prevalent in older children^1^. These unsuccessful experiences have been mainly treated with repetitive stenting, turbinate luxation or silicone nasolacrimal intubation^2^^,^^3^. Balloon dilatation is being tested in certain centers^4^. Tan et al. recommend nasolacrimal intubation when symptoms persist after two stentings, where probe or fluorescein could penetrate^5^. However, stent or irrigation attempts may not overcome obstruction, especially when dealing with fibrous or bone obstructions. Traditional dacryocystorhinostomy via endonasal endoscopy shows advantages as it avoids facial scars and is less traumatic^7^.

Our purpose is to verify if our results with endonasal endoscopic dacryocystorhinostomy in children may be considered an acceptable alternative in treatment of nasolacrimal duct obstruction in stent-resistant children.

## MATERIAL AND METHODS

The study comprised children with epiphora and ocular secretion resulting from nasolacrimal duct obstruction who were submitted to unsuccessful lacrimal stenting and were operated on for dacryocystorhinostomy via endonasal endoscopy. This procedure was approved by the Ethics Committee of our center.

Twenty-seven endonasal dacryocystorhinostomies were performed in 24 children with nasolacrimal duct obstruction from August 2000 to November 2004. All of them have been submitted to at least one lacrimal stenting. Three children were operated on both sides, in different surgical timings.

In two children who presented epiphora recurrence after external dacryocystorhinostomy, revision endoscopic surgery was performed. Admission criteria allowed them to be included in the study. Children's ages ranged from 2 to 12 years (mean: 5.7 years).

For endonasal dacryocystorhinostomy with uncinectomy, we used 4mm-diameter endoscopes, but thinner ones may also be used. We frequently use 0o angulations. Transillumination with optical fiber introduced through the lacrimal duct may confirm the lacrimal sac's location, although this is no longer routinely used by our team. Whether this approach was used in the present study was not registered.

After general anesthesia and vasoconstriction of the nasal cavity, the middle turbinate is luxated to the midline, leading to uncinate access. By means of nasal endoscopy, an uncinectomy is performed using a sickle blade. The upper part is removed with Blakesley forceps. Kerrison rongeur eliminates the mucous region and bone wall separating the lacrimal sac. When the lacrimal sac's medial wall is exposed, a lacrimal stent is introduced through the upper canaliculus, expanding or perforating the sac, which is incised with the blade. Enlargement of the sac's opening enhances perforation and removes a small portion of the medial wall with angled Blakesley. The sac's opening usually causes drainage of mucous or purulent secretion into the nasal cavity.

In our case, opening of the lacrimal sac was as small as 3mm. Silicone intubation at the end of surgery occurred with 4 children in an attempt to prevent closure. Intubation did not exceed 6 weeks. Postoperative follow-up lasted 3 months. Dacryocystorhinostomy was successful at the end of follow-up, given that the children's parents reported absence or significant reduction of epiphora and secretion.

## RESULTS

A total of 21 surgeries (77.8%) were successfully performed: after 3 months, 17 surgeries led to elimination of epiphora and secretion, while 4 surgeries resulted in significant improvement. Among children with significant reduction of symptoms, one patient improved to absence of epiphora and ocular secretion, although her lacrimal fistula remained draining. However, secretion became rare and clear.

Removal of the silicone stent was anticipated in 1 child, once it had been luxated between the lower and upper lacrimal point, and was touching the ocular globe. This prolapse was the only complication observed. In fact, this complication concerns the silicone intubation rather than the surgical technique itself.

## DISCUSSION

In children, classical dacryocystorhinostomy (external) has been avoided6, although it may be performed after 1 or 2 years of age, showing success rates over those established in adults^8^.

We believe that dacryocystorhinostomy in children requires minimal invasive techniques, such as endonasal approaches. In a series with children, Vanderveen et al. presented good outcomes in 88% of endonasal dacryocy storhinostomies^6^. Our results, regardless of being poorer, show that endonasal procedures is a valid and safe alternative. However, both our results and Vanderveen's are poorer than those obtained with external procedures, such as Barnes et al.'s outcomes (96% success rate)^9^. Wide exposure of the lacrimal sac and flap's suture obtained with external dacryocystorhinostomy lead to good outcomes, placing it in a “golden standard” position, to which other techniques are compared.

Endonasal procedures may present poorer results, but they show significant benefits: fewer traumas, less bleeding, prevention of facial scar and shorter surgery time. In children, we consider important to perform a less invasive procedure. However, not all patients are good candidates for endonasal approaches, as sometimes presence of septum deviation, anterior ethmoid cells (agger nasi), dacryocele or bleeding lead to difficult performance.

Endonasal dacryocystorhinostomy technique, as described by several authors, traditionally includes the use of drills. However, instead, we begin osteotomy with removal of the uncinate process. Uncinectomy leads to rapid removal of the lateral bone wall along with the mucosa by means of Kerrison rongeur. This step, as a way to open the lacrimal sac, avoids drilling the bone wall or using laser^10^, shortening time of dacryocystorhinostomy and avoiding the risk of nasal septum or vestibule injury, which has been reported in some children series studies^11^.

Endonasal procedures require excellent illumination and magnification. For this surgical approach, we use rigid endoscopes. Endonasal procedures using surgical microscope are conducted by some teams^12^, but use of microscope for endonasal surgery has not got as popular as the use of rigid endoscopes.

Endonasal dacryocystorhinostomy combines high success rates with less traumatic technique as compared with the external approach. In our Service, when dacryocystorhinostomy is indicated for children, we initially consider using endonasal procedure for the majority of patients, leaving the external approach for a second surgical time, for unsuccessful cases.

## CLOSING REMARKS

77.8% success rate in our surgeries, combined with minimal complication incidence, proves that endonasal endoscopic dacryocystorhinostomy is a valid and safe alternative for the treatment of stent-resistant children with nasolacrimal duct obstruction.
